# Chitosan-Based Adhesive Composite Hydrogel to Provide Antibacterial and Anti-Inflammatory Properties for Biomedical Application

**DOI:** 10.3390/gels12070580

**Published:** 2026-07-01

**Authors:** Sinuo Yan, Jingna Xu, Yue Shen, Chengde Liu, Xigao Jian, Zhonghai Li

**Affiliations:** 1 Dalian Medical University, Dalian 116044, China; yansinuodalian@outlook.com; 2 Department of Polymer Science & Materials, Dalian University of Technology, Dalian 116024, China; xujingna@mail.dlut.edu.cn (J.X.); yueshen@dlut.edu.cn (Y.S.); jian4616@dlut.edu.cn (X.J.)

**Keywords:** composite hydrogel, adhesive, antibacterial, anti-inflammation

## Abstract

Hydrogel dressings are critical in biomedical applications. However, developing multifunctional hydrogel dressings that combine antibacterial action, strong interfacial adhesion, and anti-inflammatory regulation remains a significant challenge. This study presents a novel chitosan-EMA-DMA hydrogel consisting of MgO and chitosan-curcumin nanoparticles. DMA endowed the hydrogel with adhesive performance, and EMA with water sorption enhanced the swelling ratio of the hydrogel which could control the release of the functional ingredient. The multifunctional composite hydrogel containing MgO and Curcumin demonstrated antibacterial activity and inflammatory regulation. The findings from the Fourier transform infrared spectrometer (FTIR), NMR, and SEM confirmed the successful material synthesis and the preparation of the composite hydrogel. The swelling ratio was enhanced by 50% after EMA was introduced. The adhesive strength of the composite hydrogel with skin tissue was enhanced to 90 kPa due to the introduction of DMA. The antibacterial study showed that Mg released from the multifunctional composite hydrogel enhanced the antibacterial efficacy of the composite hydrogel against *E. coli* and *S. aureus*. In vitro assessments revealed the biocompatibility and inflammatory regulation of the multifunctional composite hydrogel containing curcumin. This approach offers a strategic solution for wound dressing in biomedical applications.

## 1. Introduction

Composite hydrogel, owing to its three-dimensional network structure, excellent biocompatibility, tunable physicochemical properties, and ease of loading and controlled release of various bioactive molecules (e.g., antibacterial drugs, growth factors), has emerged as a highly promising material for hydrogel dressing. A composite hydrogel enables localized and sustained antibacterial effects through the loading of antibacterial components. Meanwhile, their flexibility and biomimetic characteristics contribute to maintaining mechanical matching and tissue integration at the interface, thereby demonstrating unique advantages in wound healing [1,2,3].

A composite hydrogel can be prepared by physical crosslinking and chemical crosslinking. Photocrosslinking, as a chemical crosslinking method that utilizes ultraviolet (UV) light-induced free radical polymerization, enables rapid gelation under mild conditions with controllable hydrogel thickness and crosslinking degree. It provides a feasible approach for the functionalization of medical dressing material [4]. Chitosan and its derivatives have garnered considerable attention in the biomedical field due to their inherent antibacterial activity, biodegradability, and ability to promote wound healing [5]. Methacrylated chitosan (CS-MA), as a photocrosslinkable chitosan derivative, not only retains the intrinsic biological activity of chitosan but also endows it with UV-curable capability [6].

Despite the aforementioned advantages of composite hydrogel in improving the properties of hydrogel, significant challenges remain in terms of antibacterial infection, as bacterial infection is a major cause of medical dressing failure. Current antibacterial strategies for composite hydrogel are mainly divided into two categories: one is to inhibit initial bacterial adhesion through antifouling surfaces (passive strategy), and the other is to kill adhered bacteria by releasing bactericidal agents (active strategy). Erathodiyil et al. employed polyethylene glycol (PEG)-based, zwitterionic polymer-based, or polyvinylpyrrolidone (PVP)-based hydrogels to establish a tightly bound, surface-bound, hydrated layer, acting as a protective barrier, thereby inhibiting nonspecific protein adsorption and initial bacterial attachment, achieving antifouling and bacteria-repelling effects. However, their antifouling performance is influenced by structural characteristics, including chain length and grafting density, requiring precise regulation to achieve optimal inhibitory effects [7,8,9]. Bai et al. [10] utilized tannic acid-modified hydrogels rich in catechol and pyrogallol groups to inhibit bacterial attachment by increasing bacterial cell membrane permeability and disrupting membrane stability. Although such polyphenol-based surface modifications exhibit antioxidant and antibacterial properties, their long-term stability and impact on biocompatibility still require further optimization. Cheng et al. [11] encapsulated the antimicrobial peptide HHC-36 along with silicate-based nanoparticles into a catechol-functionalized methacryloyl gelatin hydrogel, achieving bactericidal effects through the release of antimicrobial peptides while simultaneously promoting osteogenesis. However, the antibacterial activity is often short-lived, and there is a potential risk of side effects due to burst drug release. Slavin et al. [12] utilized hydrogels doped with metal nanoparticles such as silver, copper, and zinc oxide to kill bacteria through the release of metal ions that disrupt the bacterial outer membrane, generate reactive oxygen species, and inhibit DNA replication. Nevertheless, the antibacterial action of such systems is transient, and the nonspecific release of metal ions may induce cytotoxicity. Thus, single-mechanism antibacterial hydrogels struggle to cope with the complexity of the infectious microenvironment, leading to biofilm formation and chronic inflammation. A multifunctional platform integrating multiple synergistic antibacterial mechanisms is therefore required to achieve sustained antimicrobial efficacy [13,14].

In the context of antibacterial strategies, the natural product curcumin has attracted extensive attention due to its well-documented anti-inflammatory and antioxidant effects, as well as its broad-spectrum antibacterial performance. In terms of anti-inflammatory effects, curcumin primarily inhibits the activation of the NF-κB signaling pathway, thereby blocking the transcription of pro-inflammatory cytokines (such as TNF-α and IL-1β). Additionally, it suppresses the NLRP3 inflammasome and promotes macrophage polarization toward the anti-inflammatory M2 phenotype. In terms of antibacterial activity, curcumin can disrupt the bacterial cell membrane structure, increase membrane permeability, induce reactive oxygen species (ROS) generation upon light irradiation, thereby producing photodynamic antibacterial effects, with a low tendency to induce bacterial resistance. In terms of antioxidant activity, curcumin not only directly scavenges free radicals but also activates the Nrf2/ARE pathway to upregulate the expression of endogenous antioxidant enzymes (such as HO-1 and SOD), thereby alleviating oxidative stress-induced damage [15,16,17]. However, its poor water solubility, low stability, and limited bioavailability restrict its direct application. Encapsulation of curcumin into chitosan nanoparticles (Cur-CS NPs) not only improves its aqueous dispersibility and stability but also enables sustained drug release and synergistic antibacterial effects [18]. On the other hand, magnesium oxide nanoparticles (MgO NPs) [19], as an inorganic metal oxide nanomaterial, possess a unique antibacterial mechanism, disrupting bacterial cell membranes through magnesium ion release and reactive oxygen species (ROS) generation, while exhibiting favorable biocompatibility and low toxicity [20]. Studies have shown that MgO NPs can modulate the inflammatory microenvironment of wounds and promote angiogenesis and re-epithelialization [21]. The synergistic incorporation of Cur-CS NPs and MgO NPs into a hydrogel system is expected to achieve potent antibacterial and anti-inflammatory effects through the combined action of multiple mechanisms. Furthermore, the introduction of comonomers such as dopamine methacrylamide (DMA) and ectoine methacrylate (EMA) can further modulate the properties of the hydrogel. EMA exhibits a strong water absorption capacity, which originates from its tetrahydropyrimidine ring structure: the carbonyl and amino groups on the ring can bind water molecules through hydrogen bonding networks, and the spatial arrangement of the cyclic framework facilitates the immobilization of water molecules, thereby imparting excellent water retention capability. The adhesion of DMA (dopamine methacrylamide) arises from the catechol groups present in its side chains. The catechol structure interacts with tissue surfaces through multiple mechanisms: binding to organic surfaces via π-π stacking or hydrophobic interactions; and under neutral to weakly alkaline conditions, being oxidized to the quinone form, which then participates in Michael addition and Schiff base reactions with biomolecules containing amine or thiol groups, achieving covalent crosslinking. This multimodal adhesion mechanism enables DMA to firmly adhere to tissue surfaces even in moist or physiological environments [22].

**Scheme 1:**
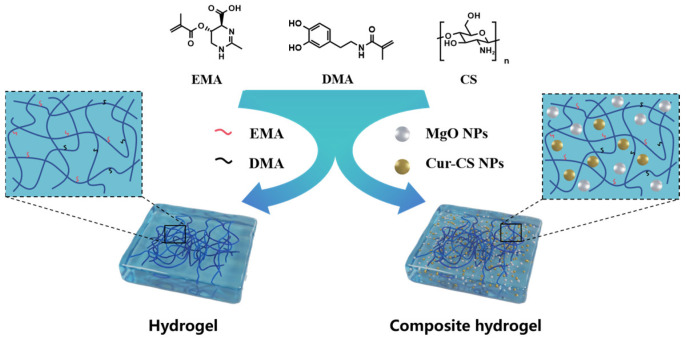
The schematic diagram of the preparation of hydrogels and the antibacterial and anti-inflammatory mechanisms of adhesive composite hydrogels.

Several recent studies have developed chitosan-based hydrogels incorporating curcumin or Mg^2+^ for wound healing [23,24]. However, most of these systems focus on either drug delivery or adhesion separately, and few combine UV-crosslinkable chitosan with both swelling-regulating EMA and adhesive DMA in a single formulation. Our work (Scheme 1) introduces three specific advances: (i) the synergistic incorporation of Cur-CS NPs and MgO NPs for sustained antibacterial and anti-inflammatory effects; (ii) a photocrosslinkable adhesive system with tunable swelling and strong tissue adhesion (~90 kPa). These features collectively distinguish our hydrogel from previously reported systems.

## 2. Results and Discussion

### 2.1. Chemical and Structural Characterization

**Figure 1:**
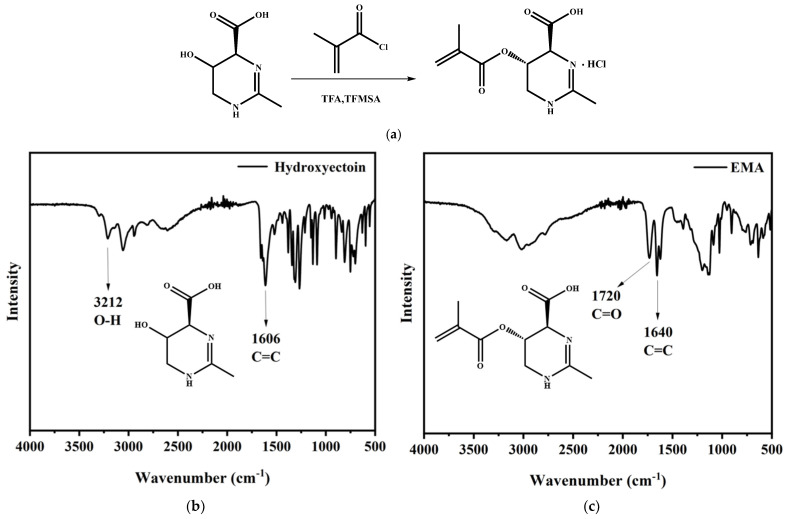
Synthesis and characterization of EMA. (**a**) synthetic procedure; (**b**) FTIR spectrum of hydroxyectoin; (**c**) FTIR spectrum of EMA.

Ectoine methacrylate (EMA) was synthesized according to a protocol established previously [25]. FTIR was used to characterize the EMA chemical structure (Figure 1b,c). Compared to the spectrum of hydroxyectoine, the broad hydroxyl peak of EMA in the range of 3200–3400 cm^−1^ decreased obviously, which suggests that the hydroxyl groups had reacted with methacryloyl chloride. A new strong absorption band appeared near 1720 cm^−1^ in the product spectrum, which was attributed to the ester carbonyl group (C=O). Furthermore, a characteristic peak corresponding to carbon–carbon double linkage (C=C) was observed at approximately 1640 cm^−1^, confirming the successful synthesis of EMA (Figure 1a).

**Figure 2:**
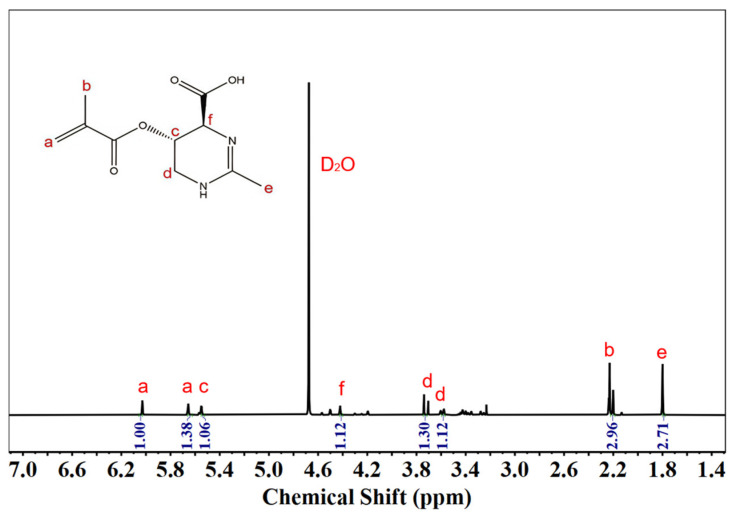
^1^H NMR spectrum of EMA.

Figure 2 shows the details of the NMR data. ^1^H NMR (600 MHz, D_2_O): Chemical shifts are observed at δ 6.05 (s, 1H), 5.67 (s, 1H), 5.58–5.57 (d, *J* = 4 Hz, 1H), 4.45 (s, 1H), 3.7–3.8 (d, *J* = 16 Hz, 1H), 3.55–3.65 (d, *J* = 16 Hz, 1H), 2.25 (s, 3H) and 1.80 (s, 3H). The successful synthesis of EMA was confirmed by ^1^H NMR spectroscopy. The spectrum exhibited characteristic signals of the methacrylate group, including two peaks at δ 6.05 and 5.67 ppm, and a signal peak at δ 2.25 ppm assigned to the methyl group conjugated to the carbon–carbon double bond.

The proton on the chiral carbon adjacent to the ester group (–COO–CH–) appeared at δ 4.45 ppm. The tetrahydropyrimidine ring was identified by the geminal coupling doublets of the diastereotopic methylene protons at δ 3.63–3.59 (d, *J* = 16 Hz) and δ 3.45–3.41 (d, *J* = 16 Hz), a methine proton doublet at δ 5.58–5.57 (d, *J* = 4 Hz), and a methyl singlet at δ 2.25 ppm. All chemical shifts and coupling constants were consistent with the proposed structure, indicating the successful preparation of the EMA monomer.

ESI-MS *m*/*z*: calculated [M+H]^+^ for EMA, 227.2286; found, 227.1135. The agreement of the measured value with the theoretical calculation further confirms the successful synthesis of EMA (Figure S1).

**Figure 3:**
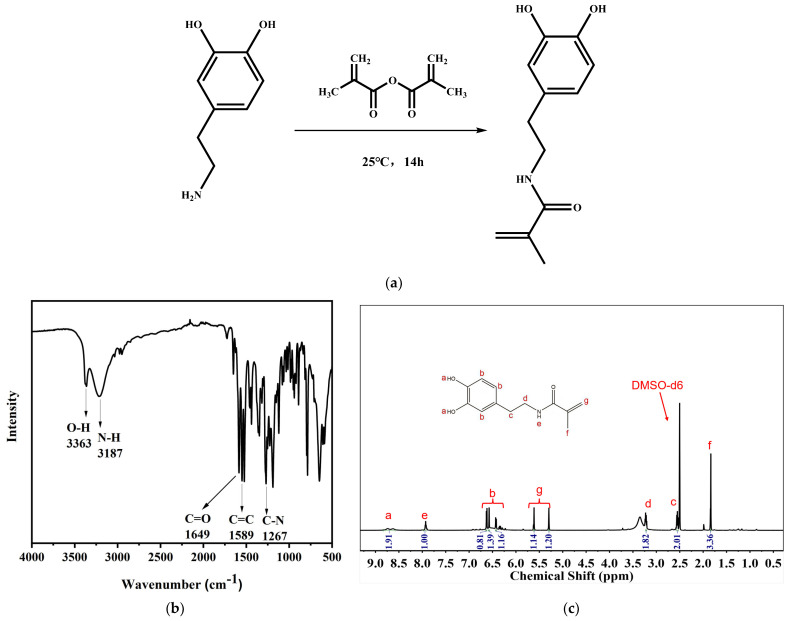
Synthesis and characterization of DMA. (**a**) synthetic procedure; (**b**) FTIR spectrum of DMA; (**c**) ^1^H NMR spectrum of DMA.

The FT-IR spectrum of DMA (Figure 3b) showed typical absorption bands at 3363 cm^−1^ and 3187 cm^−1^, which were assigned to the stretching vibrations of hydroxyl (–OH) and amino (–NH) groups, respectively, indicating the retention or transformation of the relevant functional groups from the starting materials. A strong absorption band at 1649 cm^−1^ was attributed to the stretching vibration of carbonyl (C=O), confirming the successful introduction of the carbonyl group. The absorption peak observed at 1589 cm^−1^ was attributed to the carbon–carbon double bond (C=C) stretching mode, demonstrating the presence of the expected alkene structure in the product. In addition, the characteristic peak at 1267 cm^−1^ corresponded to the carbon–nitrogen (C–N) stretching vibration, providing additional evidence for the formation of nitrogen-containing functional groups. The sequential appearance of all these characteristic functional-group absorption peaks, which are fully consistent with the predicted structure of DMA, collectively confirms the successful synthesis of the target product DMA.

^1^H NMR spectra of DMA. ^1^H NMR (600 MHz, DMSO(d6)): δ 8.75 (singlet, 1H), 8.63 (singlet, 1H), 7.90 (multiplet, 1H), 6.30–6.70 (multiplet, 3H), 5.65 (singlet, 1H), 5.25 (singlet, 1H), 3.20–3.60 (triplet, *J* = 8 Hz, 2H), 2.55–2.65 (triplet, *J* = 8 Hz, 2H), 1.80 (singlet, 3H). The successful synthesis of DMA was confirmed by ^1^H NMR spectroscopy (Figure 3c). The methacrylamide group was identified by two terminal =CH_2_ singlets at δ 5.65 and 5.25 ppm, along with a methyl singlet at δ 1.80 ppm. All chemical shifts and coupling constants were consistent with the proposed molecular structure of DMA, confirming the successful preparation of the monomer (Figure 3a).

ESI-MS *m/z*: [M+H]^+^ calculated for: [M+H]^+^ calculated for DMA 222.2518; found 222.1124. The agreement of the measured value with the theoretical calculation further confirms the successful synthesis of DMA (Figure S2).

**Figure 4:**
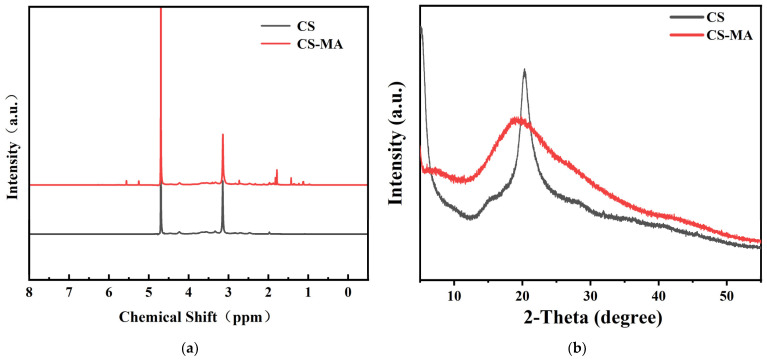
^1^H NMR (**a**) spectra and XRD patterns (**b**) of CS and CS-MA.

The modified chitosan was identified by ^1^H NMR spectroscopy before and after the modification, as shown in Figure 4a. In the NMR spectrum, distinct vinyl proton peaks appeared in the range of 5–6 ppm, and the methyl hydrogen signal at 1.85 ppm was markedly enhanced after modification, indicating that the methyl group from methacrylic anhydride had been successfully grafted onto the chitosan backbone (Figure S3).

The XRD patterns of chitosan and methacrylic anhydride-modified chitosan are shown in Figure 4b. Both pristine and modified chitosan exhibit amorphous structures. These results indicate the successful synthesis of double-bond-modified chitosan, which can address the poor solubility of chitosan, confer UV reactivity, and thereby provide a foundation for hydrogel preparation.

**Figure 5:**
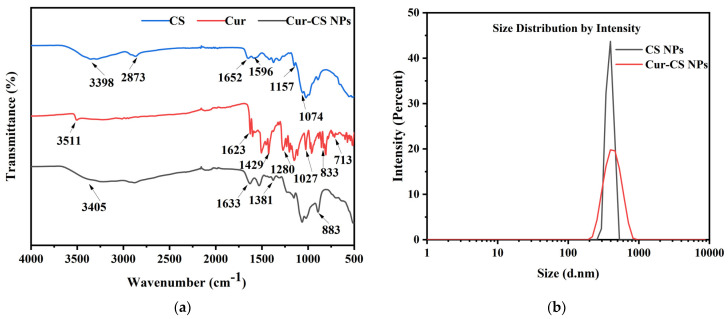
Chemical structure and nanoparticle size distribution. (**a**) FTIR spectra of chitosan, curcumin, and Cur-CS nanoparticles; (**b**) Particle size distribution profiles of CS NPs and Cur-CS NPs.

In Figure 5a, for CS, the absorption peak at 3398 cm^−1^ was assigned to the stretching vibration of O-H bonds, the peak at 2873 cm^−1^ to the stretching vibration of C-H bonds, the peak at 1652 cm^−1^ to the stretching vibration of the amide I band, the peak at 1596 cm^−1^ to the stretching vibration of the amide II band, the peak at 1157 cm^−1^ to the stretching vibration of C-O-C, and the peak at 1074 cm^−1^ to the stretching vibration of C-O bonds. For Cur, the absorption peak at 3511 cm^−1^ was ascribed to the stretching vibration of O-H bonds, the peak at 1623 cm^−1^ to the stretching vibration of C=O bonds, the peak at 1429 cm^−1^ to the bending vibration of C-H bonds, the peak at 1280 cm^−1^ to the stretching vibration of C-O bonds, the peak at 1027 cm^−1^ to the stretching vibration of C-O-C, and the peaks at 833 and 713 cm^−1^ to the vibrations of the benzene ring. In the Cur-CS NPs, characteristic peaks of both Cur and CS were observed, confirming the successful loading of Cur into the CS nanoparticles.

The zeta potential and polydispersity index (PDI) of CS NPs and Cur-CS NPs were determined using a nanoparticle size and zeta potential analyzer. As presented in Figure 5b and Table 1, the mean particle size of CS NPs was 389.7 ± 44.6 nm, while that of Cur-CS NPs slightly increased to 436.9 ± 115.7 nm. This increase in particle diameter may be assigned to the loading of a certain amount of curcumin (Cur) onto the CS NPs. The Zeta potentials of both chitosan nanoparticles and Cur-loaded chitosan nanoparticles were positive, measured at 5.2 ± 3.6mV and 9.7 ± 4.7mV, respectively, indicating that the synthesized nanoparticles possessed good stability. The PDI values of chitosan nanoparticles and Cur-loaded chitosan nanoparticles were relatively low, at 0.406 ± 0.24 and 0.23 ± 0.12, respectively. The slightly decreased dispersity of Cur-CS NPs compared to CS NPs may be due to the strong hydrophobicity and poor solubility of curcumin.

### 2.2. Preparation and Characterization of Hydrogel

**Figure 6:**
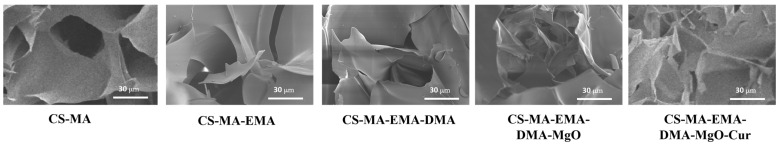
SEM images of the hydrogels.

Scanning electron microscopy (SEM) was utilized to investigate the microstructure of freeze-dried hydrogels, as shown in Figure 6. In the cross-sectional images of each hydrogel, a representative porous structure can be observed. Moreover, the pore size exhibits a gradually decreasing trend (Table S1), which is attributed to the incorporation of DMA, MgO, Cur-CS NPs, and other substances that progressively reduce the pore size of the hydrogels. The crosslinked polymer network exhibits a complex structure with interpenetrating macropores and micropores. In fact, this structure facilitates the efficient exchange of nutrients and metabolic waste products in vivo, thereby creating a suitable environment for cell proliferation, differentiation, and inward migration.

**Figure 7:**
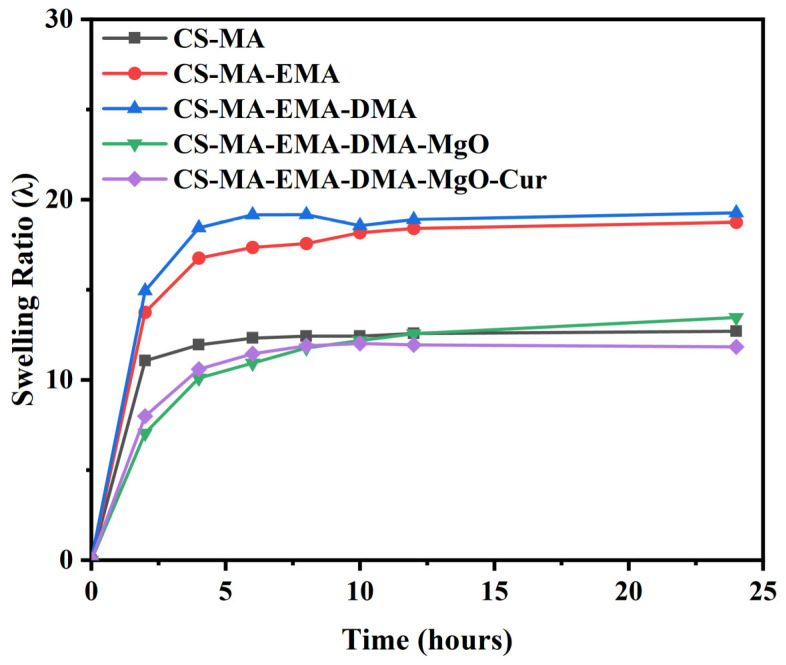
Swelling kinetics of the hydrogels in PBS.

The swelling characteristics of hydrogels are crucial for nutrient transport, oxygen exchange, and water exchange. Excessive swelling can compromise the mechanical properties of the hydrogel and compress surrounding tissues, thereby impeding normal metabolism and ultimately affecting tissue regeneration. Conversely, insufficient swelling ratios prevent the hydrogel from adequately absorbing surface moisture from tissues, limiting its adhesive performance. To evaluate this, the swelling properties of five hydrogels—CS-MA, CS-MA-EMA, CS-MA-EMA-DMA, CS-MA-EMA-DMA-MgO, and CS-MA-EMA-DMA-MgO-Cur—were assessed by immersing them in PBS buffer until swelling equilibrium was reached, with the results presented in Figure 7. The swelling behavior was characterized based on the obtained swelling curves. All hydrogels, initially of the same dimensions, exhibited a marked increase in swelling ratio upon immersion in PBS. Over time, the swelling ratios gradually increased for all formulations, reaching equilibrium in PBS buffer after approximately 10 h. The CS-MA-EMA-DMA hydrogel network displayed the highest swelling capacity, attributable to the presence of ectoine methacrylate (EMA), which possesses strong hydration capability, and hydrophilic dopamine methacrylamide (DMA). Removal of DMA (CS-MA-EMA) resulted in a slight decrease in hydrophilicity and, consequently, a lower swelling ratio. The pure CS-MA homopolymer network, lacking the strong hydration effect of EMA and exhibiting higher chain rigidity, showed a further reduced swelling ratio. Upon the introduction of MgO, the released Mg^2+^ ions formed strong metal-ligand coordination crosslinks with the catechol groups of DMA, significantly increasing the physical crosslinking density and thereby substantially suppressing swelling. Finally, with the further addition of hydrophobic curcumin (Cur), additional physical crosslinks were introduced via hydrophobic interactions and π-π stacking with the aromatic structures of DMA. Moreover, curcumin occupies free volume within the network and repels water molecules. This dual inhibitory effect, superimposed on the Mg^2+^-mediated coordination crosslinking, results in the most compact network structure, consequently yielding the lowest swelling ratio among all tested hydrogels.

The adhesion ability of the hydrogels to soft tissues was characterized using a porcine skin lap shear test. The hydrogel formed a bond with the porcine skin tissue surface with an adhesion area of approximately 10 × 10 mm^2^. According to the lap shear test data, the adhesion strength of the hydrogel was significantly improved after the incorporation of DMA, compared to the CS-MA group. The CS-MA-EMA-DMA-MgO-Cur hydrogel achieved a maximum adhesion strength of 0.092 MPa, which exhibited a three-fold increase compared with the CS-MA group (Figure S4). This enhancement is attributed to the catechol groups of DMA that can generate hydrogen bonds, coordination bonds, and covalent bonds with tissue surfaces (e.g., proteins, polysaccharides), thereby endowing the hydrogel with robust wet-tissue adhesion capability. Compared with previously reported mussel-inspired adhesive hydrogels, the adhesion strength of our composite hydrogel (~90 kPa) is comparable to or higher than several representative systems. For instance, a double-crosslinking chitosan-catechol hydrogel developed by Zhao et al. exhibited an adhesion strength of approximately 45.6 kPa on porcine skin [26]; a chitosan-catechol derivative optimized by KIST achieved a lap shear strength of up to 64.8 ± 5.7 kPa [27]. The superior adhesion of our system likely originates from the synergistic effect of DMA’s catechol groups and the enhanced crosslinking density provided by Mg^2+^ coordination, which distributes stress more effectively at the tissue interface. These results indicate that the hydrogel possesses favorable adhesion properties.

**Figure 8:**
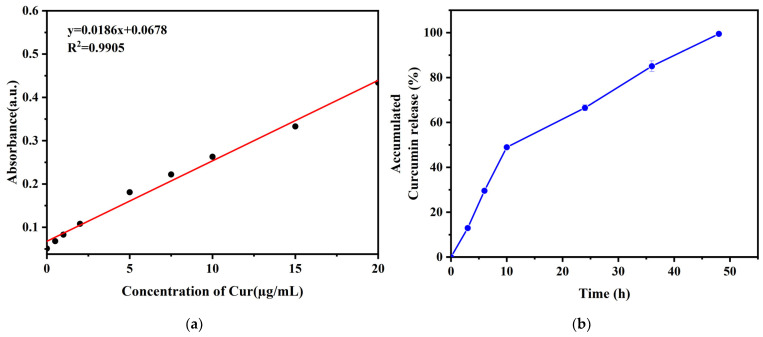
**In vitro curcumin release study.** (**a**) Calibration curve of curcumin in PBS at 425 nm; (**b**) cumulative release of curcumin from the CS-MA-EMA-DMA-MgO-Cur hydrogel over 48 h.

The cumulative release curve of curcumin from the CS-MA-EMA-DMA-MgO-Cur disks in PBS demonstrated a sustained release behavior (Figure 8a,b). In the initial stage (0–10 h), a relatively rapid release was observed, followed by a gradually slowed and more stable release phase. By 48 h, the cumulative release reached approximately 99%. This release pattern indicates that the prepared composite material exhibits favorable sustained-release capability for curcumin, which is beneficial for achieving long-term drug delivery.

**Figure 9:**
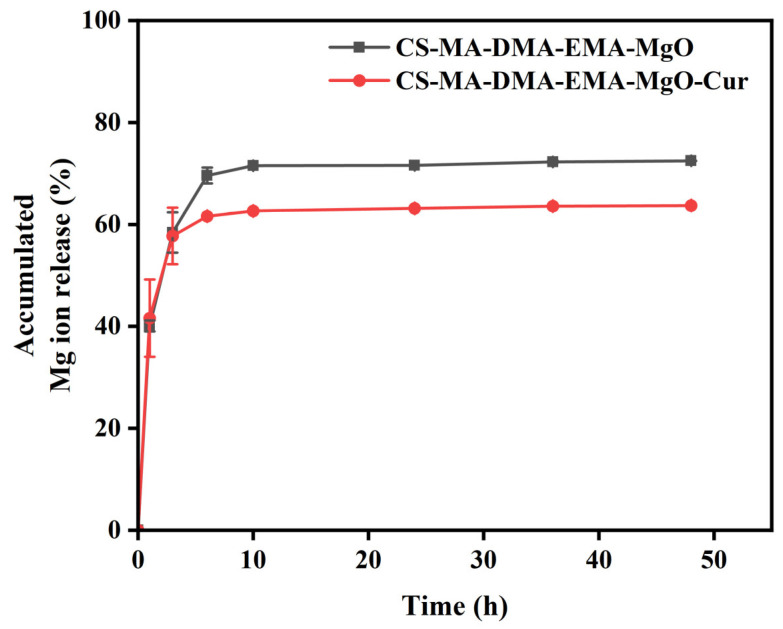
Curves of accumulated Mg ion release.

The Mg^2+^ release behavior (Figure 9) showed that both groups of hydrogels exhibited release of the majority of magnesium ions during the initial stage, with the release rate of Mg^2+^ decreasing and stabilizing as the soaking time increased. The CS-MA-EMA-DMA-MgO hydrogel achieved a rapid release within 8 h, reaching a cumulative release rate of approximately 72%, after which it stabilized. In contrast, the curcumin-loaded CS-MA-EMA-DMA-MgO-Cur hydrogel exhibited a reduced cumulative release rate at the plateau phase, reaching approximately 63%. This difference is primarily attributed to the fact that Cur-CS nanoparticles effectively delay the hydration of MgO and the diffusion of Mg^2+^ through physical barrier effects, electrostatic interactions, and competitive crosslinking, thereby reducing both the total release amount and release rate of Mg^2+^ and achieving controlled release of magnesium ions.

**Figure 10:**
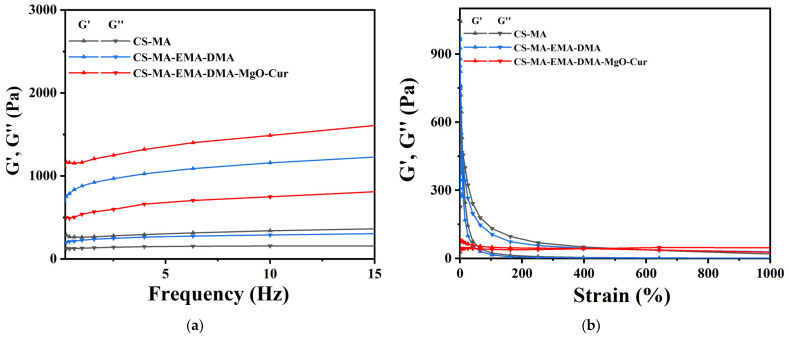
Rheological properties of hydrogel: (**a**) The frequency sweep test; (**b**) The strain sweep test.

During the oscillation frequency sweep (Figure 10a), the storage modulus (G′) was higher than the loss modulus (G″) for all hydrogels. At 1 Hz, the CS-MA-EMA-DMA-MgO-Cur hydrogel exhibited a G′ of 1164 Pa and a G″ of 538.9 Pa. Smooth curves were observed within the range of 0.1–15 Hz, indicating well-maintained hydrogel structures and elastomeric characteristics. The oscillation strain sweep results showed that the CS-MA-EMA-DMA-MgO-Cur hydrogel exhibited an intersection point between G′ and G″ within the strain range of 0.1–1000% (Figure 10b). Among all formulations, the CS-MA-EMA-DMA-MgO-Cur hydrogel demonstrated the highest limiting strain, approaching 500%, which notably exceeded the maximum strain capacity of human skin [28]. These results indicate that the hydrogel possesses good viscoelasticity and resistance to deformation.

**Figure 11:**
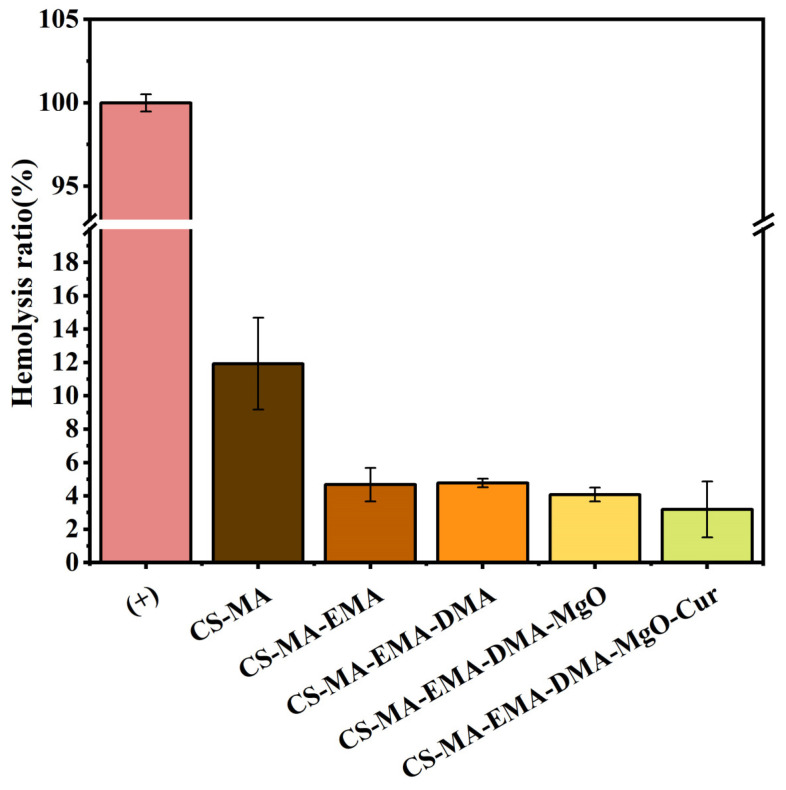
Hemolysis ratio of CS-MA, CS-MA-EMA, CS-MA-EMA-DMA, CS-MA-EMA-DMA-MgO, and CS-MA-EMA-DMA-MgO-Cur.

For biomaterials and medical devices contacting blood, hemocompatibility is an essential property. These materials shall avoid unfavorable interactions with blood constituents and prevent the activation and destruction of blood cells and related components. Pristine CS-MA hydrogel exhibited a hemolysis ratio of 11.9%, exceeding the 5% non-hemolytic threshold and indicating inherent incompatibility with blood. Incorporation of EMA into the network (CS-MA-EMA) substantially reduced hemolysis to 4.7%, falling within the acceptable non-hemolytic range. Supported by the 50% enhancement in the swelling ratio observed upon EMA incorporation, we hypothesize that EMA provides a more hydrated microenvironment that may mitigate erythrocyte membrane disruption. Subsequent modifications incorporating DMA, MgO nanoparticles, and curcumin maintained hemolysis ratios of 4.8%, 4.1%, and 3.2%, respectively, all remaining below the critical 5% limit (Figure 11). Collectively, these results demonstrate that systematic functionalization effectively imparts hemocompatibility suitable for topical wound dressings. To explore its broader potential in implantable applications in the future, more comprehensive blood compatibility assessments will be systematically investigated.

**Figure 12:**
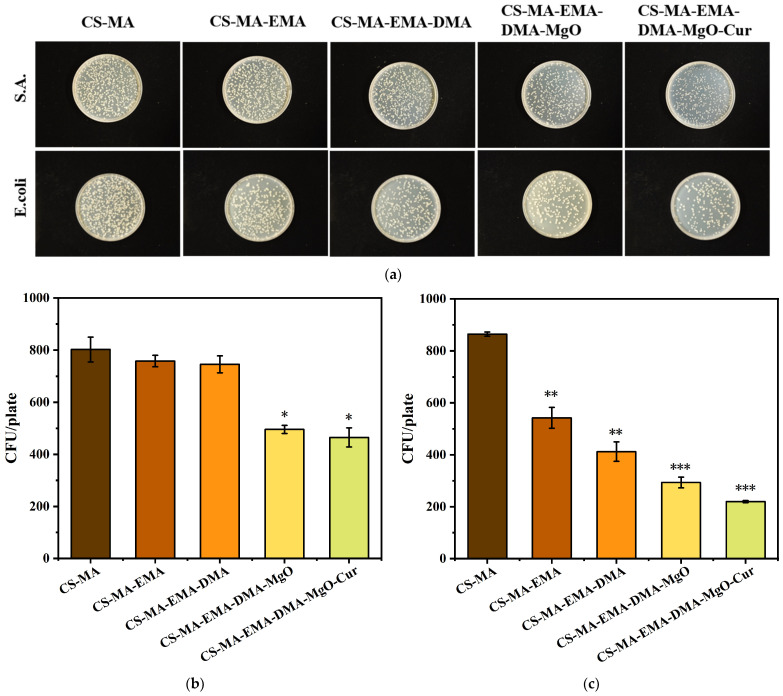
(**a**) Colonies of *S. aureus* and *E. coli* on the agar plates after sample co-culture with bacterial Suspension, and corresponding quantitative analysis of (**b**) *S. aureus* colonies and (**c**) *E. coli* colonies. ANOVA was utilized to ascertain statistical significance; significance was denoted by * *p* < 0.05, high significance by ** *p* < 0.01, and very high significance by *** *p* < 0.001.

The agar plate counting assay demonstrated a pronounced stepwise enhancement in the antibacterial efficacy of the composite hydrogel against *E. coli* and *S. aureus* upon sequential introduction of the modifying components. As quantified and statistically confirmed in Figure 12a–c, relative to the pristine CS-MA substrate, the incorporation of EMA and DMA conferred more sensitive inhibition toward *E. coli*, a phenomenon attributable to differential interactions with its comparatively thinner peptidoglycan cell wall layer. Subsequent doping with MgO led to a marked decline in the survival rates of both bacterial species. Based on established literature, this bactericidal effect is driven by MgO nanoparticles physically disrupting membrane integrity and generating H_2_O_2_ to induce severe oxidative stress [20]. Curcumin further amplifies this efficacy by structurally damaging bacterial lipid bilayers, causing fatal intracellular leakage [29]. Ultimately, the CS-MA-EMA-DMA-MgO-Cur composite hydrogel achieved optimal broad-spectrum antibacterial performance. Taken together, the superior antibacterial properties of this hydrogel arise from a profound synergistic interplay among the active, multi-target bactericidal actions of its internal constituents, underscoring its considerable potential for anti-infective applications in topical wound dressings.

**Figure 13:**
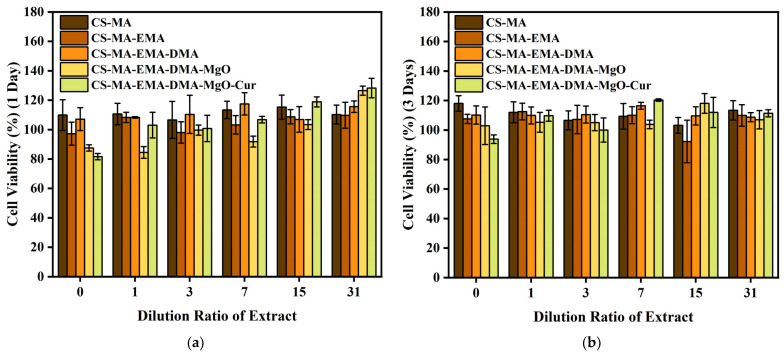
Viability of MC3T3-E1 cells cultured with CS-MA, CS-MA-EMA, CS-MA-EMA-DMA, CS-MA-EMA-DMA-MgO, and CS-MA-EMA-DMA-MgO-Cur extracts at (**a**) 1 day and (**b**) 3 days.

As the wound dressing, biosafety must be the primary consideration. The biocompatibility of five materials—CS-MA, CS-MA-EMA, CS-MA-EMA-DMA, CS-MA-EMA-DMA-MgO, and CS-MA-EMA-DMA-MgO-Cur—was evaluated using the MTT assay, as shown in Figure 13a,b. All experiments were performed with three replicates (*n* = 3). The results demonstrated that none of the five materials, namely CS-MA, CS-MA-EMA, CS-MA-EMA-DMA, CS-MA-EMA-DMA-MgO, and CS-MA-EMA-DMA-MgO-Cur, induced any significant cytotoxicity.

**Figure 14:**
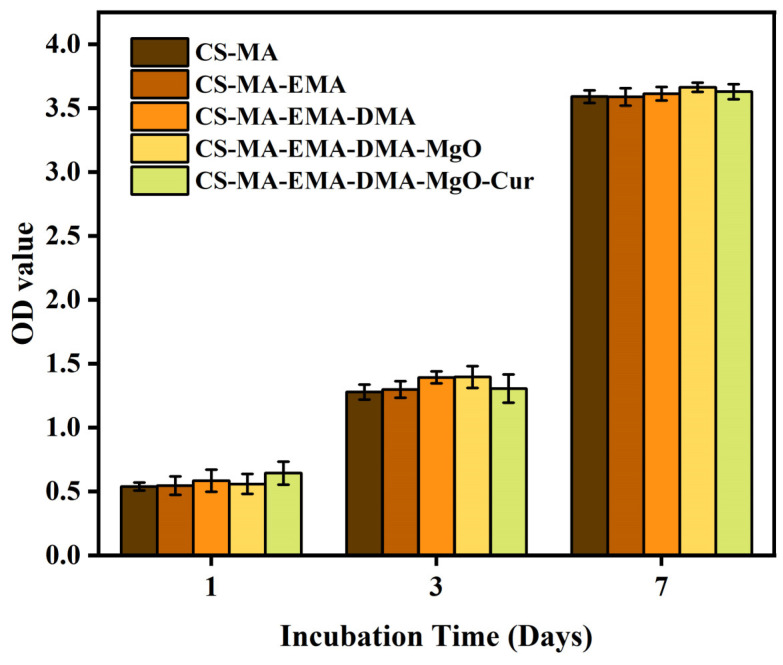
CCK-8 analysis of MC3T3-E1 cells cultured with CS-MA, CS-MA-EMA, CS-MA-EMA-DMA, CS-MA-EMA-DMA-MgO, and CS-MA-EMA-DMA-MgO-Cur extracts for 1, 3, and 7 days.

Furthermore, the CCK-8 assay was employed to further quantitatively analyze the cell proliferation. While the MTT assay served as an initial screening for acute mitochondrial metabolic activity (days 1 and 3), the CCK-8 assay offers higher sensitivity and lower cell toxicity, making it suitable for longer-term proliferation assessment up to 7 days. The complementary use of both assays provides a more comprehensive evaluation of cytocompatibility. As shown in Figure 14, the cells were cultured with the samples for 1, 3, and 7 days. The cells increased exponentially from 1 to 7 days, and the cell viability was maintained at about 100%, which indicated that the cells grew well with the samples.

**Figure 15:**
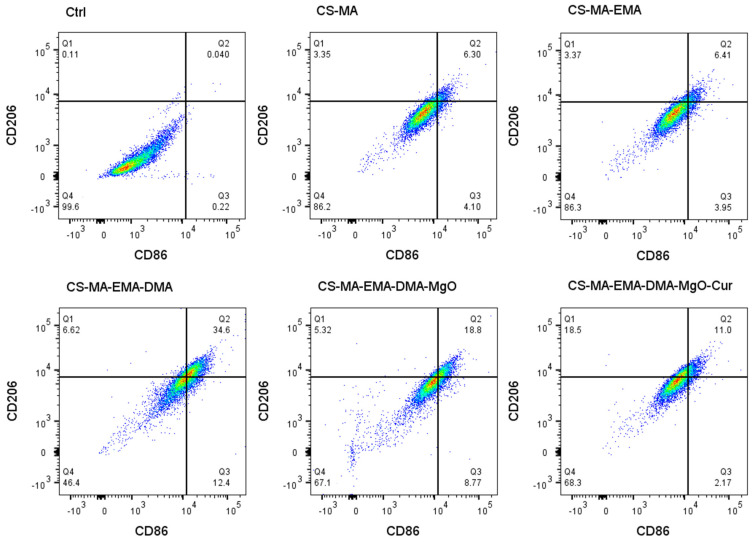
Scatter plots depicting the RAW264.7 cellular surface markers CD86 (indicative of M1 macrophages) and CD206 (indicative of M2 macrophages) were analyzed via flow cytometry.

Flow cytometry analysis (Figure 15) was performed using an unstained control to set the gates (99.6% double-negative cells in Q4). The gating strategy was as follows: cells were first gated on forward/side scatter to exclude debris, followed by single-cell gating. Quadrants were defined based on the unstained control, with Q4 representing double-negative (CD86^−^CD206^−^), Q3 representing M1 (CD86^+^CD206^−^), Q1 representing M2 (CD86^−^CD206^+^), and Q2 representing double-positive (CD86^+^CD206^+^) cells. Experiments were performed in triplicate (n = 3). Among the hydrogel-treated groups, the curcumin-loaded hydrogel (CS-MA-EMA-DMA-MgO-Cur) showed the highest M2 population (18.5% in Q1) and the lowest M1 population (2.17% in Q3), along with 11.0% double-positive cells (Q2) and 46.4% double-negative cells (Q4). In contrast, the DMA-containing hydrogel without curcumin (CS-MA-EMA-DMA) exhibited a markedly elevated double-positive fraction (34.6% in Q2) but a much lower M2 percentage (6.62%), indicating that DMA alone promotes an intermediate/transitional state rather than full M2 polarization. The subsequent addition of MgO (CS-MA-EMA-DMA-MgO) reduced the double-positive cells to 18.8% without further increasing M2, whereas curcumin was essential to drive the shift toward the M2 phenotype. These results demonstrate that curcumin, not the other modifications, is the key functional component that repolarizes macrophages toward an M2-dominant state under inflammatory conditions.

## 3. Conclusions

In summary, this study successfully designed and constructed a novel chitosan-based composite hydrogel. The hydrogel possesses excellent adhesion properties, antibacterial performance, immunomodulatory potential (promoting M2 macrophage polarization), and biocompatibility. An adhesive composite hydrogel was successfully fabricated via UV-initiated free radical polymerization.

The combination of nano-magnesium oxide particles and curcumin enables the composite hydrogel to achieve an antibacterial rate of over 80% against Staphylococcus aureus and Escherichia coli. Cytocompatibility tests confirmed that all hydrogel groups exhibited no obvious cytotoxicity. Immunoflow cytometry results indicated that the CS-MA-EMA-DMA-MgO-Cur hydrogel significantly promoted macrophage polarization toward the M2 phenotype (increased CD206 expression) while reducing the M1 phenotype (decreased CD86 expression), suggesting a potential to modulate the inflammatory response toward a pro-regenerative state. This multifunctional hydrogel offers a strategic solution for wound dressings in biomedical applications.

## 4. Materials and Methods

### 4.1. Materials

Chitosan was bought from Xilong Scientific Co., Ltd. (Shantou, China). Dopamine was commercially available from Qifei Pharmaceutical Chemical Co., Ltd. (Tianmen, China). Hydroxyectoine was purchased from Shanghai Titan Scientific Co., Ltd (Shanghai, China). Methyl alcohol (MeOH) and n-hexane were purchased from Tianjin Jindong Tianzheng Fine Chemical Reagent Factory (Tianjin, China). Trifluoroacetic acid, methacryloyl chloride, tetrahydrofuran, sodium tripolyphosphate (TPP), anhydrous ethanol, and Magnesium oxide (MgO) (ca. 50 nm in diameter) were purchased from Shanghai Macklin Biochemical Co., Ltd. (Shanghai, China). Methacrylic anhydride (MA), trifluoromethanesulfonic acid, sodium tetraborate, sodium bicarbonate, ethyl acetate, anhydrous magnesium sulfate, and curcumin (Cur) were purchased from Shanghai Aladdin Biochemical Technology Co., Ltd. (Shanghai, China). Common Gram-positive and Gram-negative bacteria were used in this experiment. *S. aureus* (CMCC 26003) and *E. coli* (ATCC 25922) were used as representatives. Rabbit whole blood is obtained from Shanghai yuanye Bio-Technology Co., Ltd., Shanghai, China. The remaining reagents, all of analytical grade purity, were employed in their received state for the experiments.

### 4.2. Experimental Methods

#### 4.2.1. Synthesis of EMA, DMA, and CS-MA

EMA was synthesized according to the previously reported method [25,30]. Typically, 2 g of hydroxyectoine was dispersed in 8 mL of trifluoroacetic acid, and the mixture was stirred at 0 °C until the solute was fully dissolved. Afterward, 197 μL of trifluoromethanesulfonic acid and 2.44 mL of methacryloyl chloride were sequentially introduced into the solution, followed by continuous stirring for 2 h. Subsequently, 30 mL of diethyl ether was dropwise added to the above reaction system in ice bath conditions (0 °C), and a white solid precipitate was generated. The obtained crude product was further purified via recrystallization using a mixed solvent of methanol (MeOH) and diethyl ether, and rinsed thoroughly with diethyl ether to finally acquire the EMA monomer (yield: 83%).

A total of 1 g of sodium tetraborate, 0.4 g of sodium bicarbonate, and 10 mL of deionized water were stirred under a nitrogen atmosphere for 20 min. A total of 0.5 g of dopamine hydrochloride was subsequently introduced. After thorough mixing, 0.48 mL of methacrylic anhydride solution and 3 mL of tetrahydrofuran were slowly added dropwise to the above aqueous solution. After adjusting the pH of the mixture to 8 or above, the reaction was incubated at room temperature for 14 h, yielding a gray-pink slurry-like solution. The solution was extracted with ethyl acetate, and the upper organic layer was then separated out. The remaining aqueous layer was centrifuged at 4000 rpm for 3 min. A 6 N hydrochloric acid solution was added to the supernatant until precipitation occurred. Ethyl acetate was further added to extract the supernatant, which was then dried over an appropriate amount of anhydrous magnesium sulfate. Centrifugation was performed at 4000 rpm for 5 min on the solution, and the resulting supernatant was precipitated into n-hexane at a ratio of 1:10 (*v*/*v*), yielding a gray-white crude product. The crude product was redissolved in 7 mL of ethyl acetate and reprecipitated into n-hexane at the same ratio to obtain a white final product (yield: 90%) [31].

Into 100 mL of 0.1 mol/L acetic acid, 1.5 g of chitosan powder was added and fully dissolved in the solution under stirring for 12 h until completely dissolved. The solution was then placed in a 40 °C oil bath. A total of 6 mL of methacrylic anhydride was slowly added dropwise to the reaction system, and the reaction was allowed to proceed under light-protected conditions at 40 °C for 12 h to achieve efficient grafting of methacrylic anhydride. Upon completion of the reaction, the reaction mixture was transferred using a dropper into a dialysis bag with a molecular weight cutoff of 14,000 Da, and dialyzed against deionized water at 40 °C for one week. During the dialysis process, a white precipitate gradually formed and settled at the bottom of the bag. The clear supernatant was collected via centrifugation, and subsequently lyophilized using a freeze dryer to obtain methacrylic anhydride-modified chitosan (CS-MA) (Degree of Substitution: 50%). The obtained CS-MA samples were stored at −20 °C for subsequent experiments [32,33].

#### 4.2.2. Characterization of EMA, DMA, and CS-MA

Fourier transform infrared spectroscopy (FT-IR) was performed with a Nicolet 7199 spectrometer (Thermo Fisher Scientific, Madison, WI, USA). The samples were ground and mixed with potassium bromide (KBr), followed by compression into pellets. The spectra were then measured using an advanced the wavenumber was set between 4000 and 500 cm^−1^ for Fourier transform infrared characterization.

The changes in characteristic functional groups of the material before and after modification were analyzed by proton nuclear magnetic resonance (^1^H NMR) spectroscopy (AVANCE NEO 600M, Bruker, Karlsruhe, Germany). Approximately 10 mg of each sample, both before and after modification, was dissolved in deuterium oxide (D_2_O). For chitosan and modified chitosan, a mixture of deuterium oxide (D_2_O) containing 10 μL of deuterated trifluoroacetic acid (TFA-d) was used as the solvent. Tetramethylsilane (TMS) was employed as the internal standard. The ^1^H NMR spectra were recorded at an observation frequency of 400 MHz at room temperature.

The molecular weight was analyzed by Linear Ion Trap-High Resolution Liquid Chromatography-Mass Spectrometry (LTQ Orbitrap XL; GC-MS, 8890-5977B, Agilent Technologies, CA, USA).

X-ray diffraction (XRD) patterns were recorded on the Thermo Scientific ESCALAB 250Xi spectrometer (Waltham, MA, USA) using Cu Kα radiation (λ = 1.5406 Å) at 40 kV and 40 mA. A 2θ scanning range of 5–80° and a step size of 0.02° were applied for sample detection.

#### 4.2.3. Preparation and Characterization of Cur-CS NPs

The Cur-CS nanoparticle samples were synthesized by the ionic crosslinking method [18]. Specifically, 50 mg of CS was accurately weighed using an electronic balance and fully dissolved in 50 mL of aqueous acetic acid (2% *w*/*v*). The mixture was ultrasonicated in an ice bath until the CS was completely dissolved, yielding a solution concentration of 1 mg/mL.

Following the same procedure, 1000 mg of TPP was accurately weighed and added to 50 mL of deionized water and dissolved, followed by ultrasonication until the solution became clear and transparent, resulting in a TPP solution with a concentration of 2% *w*/*v*. Under light-protected conditions, after precise weighing, a certain amount of Cur was dissolved in anhydrous ethanol, forming a 10 mL Cur solution at 1.5 mg/mL. The Cur solution was slowly dripped into a 10 mL CS solution, with magnetic stirring kept at a constant speed. Subsequently, the TPP solution was slowly added dropwise using a syringe until a pale yellow opalescence appeared. Stirring was continued for another 10 min, and the opalescence persisted. The resulting suspension was then transferred to a centrifuge pre-cooled to 4 °C, and a 15 min spinning process was applied to the mixture at 12,000 rpm. The upper liquid phase was abandoned, and the precipitate was washed three times with 10% aqueous methanol solution. After 24 h of freeze-drying, the collected precipitate turned into Cur-CS NPs, which were subsequently preserved in the dark.

Nanoparticle size and Zeta potential testing were carried out using Nanoparticle Size and Zeta Potential Analyzer (Zetasizer; Nano ZS90, Malvern, UK). Appropriate amounts of blank CS NPs and Cur-CS NPs were dispersed in deionized water. An appropriate volume of each dispersion was transferred using a micropipette into the cuvettes provided with the nanoparticle size and Zeta potential analyzer. All operations were carried out at 25 °C to obtain the size distribution, Zeta potential values, as well as their polydispersity index (PDI) [34].

#### 4.2.4. Preparation of the Multifunctional Hydrogel

A hydrogel precursor solution was prepared by dissolving 10 wt% CS-MA, 8 wt% EMA, 0.2 wt% DMA, 0.2 wt% MgO, 0.25 mg/mL curcumin-loaded nanoparticles (Cur-CS NPs), and 1 wt% photoinitiator 2959 (Irgacure 2959), with the balance being water. The detailed composition of the hydrogel formulation is presented in Table S2. After thorough dissolution, the precursor solution was injected into a mold and cured under a UV oven for 3 min. The resulting hydrogels were designated as CS-MA, CS-MA-EMA, CS-MA-EMA-DMA, CS-MA-EMA-DMA-MgO, and CS-MA-EMA-DMA-MgO-Cur, respectively.

#### 4.2.5. Hydrogel Morphology and Swelling Ratio Measurement

The hydrogel morphology was observed and imaged using a tungsten filament scanning electron microscope (SEM) (Hitachi, S-4800, Ibaraki Prefecture, Tokyo, Japan). Prior to measurement, the samples were freeze-dried and sputter-coated with platinum for 60 s.

To investigate the swelling behavior of the hydrogel samples, the freeze-dried samples were first accurately weighed to obtain their dry mass (W_d_). Subsequently, the samples were immersed in phosphate-buffered saline (PBS, pH 7.4) at 37 °C. At predetermined time intervals, the swollen samples were removed, and their wet mass (W_t_) was recorded after gently blotting away the excess surface water with filter paper. The swelling ratio (SR) was calculated as [35,36]:

(1) $$SR\left(\%\right)=\frac{W_{t}-W_{d}}{W_{d}}\times 100\%$$

#### 4.2.6. Adhesion Study

Following the test method, fresh porcine skin was first cut into rectangles of 30 mm × 10 mm and immersed in PBS buffer, then stored at 4 °C [37]. Prior to testing, excess liquid on the surface of the porcine skin was removed using filter paper. Subsequently, the hydrogel was adhered to the tissue side of the porcine skin with a bonding area of 10 mm × 10 mm, while finger pressure was applied to the bonding site for approximately 20 s. The adhesive strength of the hydrogel was then measured using a universal testing machine at a tensile rate of 50 mm/min with a 100 N load cell under room temperature conditions [38].

#### 4.2.7. Curcumin and Mg Release Measurement

Curcumin (Cur) solutions were prepared in PBS at concentrations of 0.5, 1, 2.5, 5, 7.5, 10, 15, and 20 μg/mL. The blank control group was prepared with pure PBS. All solutions were measured for absorbance at 425 nm using a UV-Vis spectrophotometer (Cary 5000, Agilent Technologies, Santa Clara, CA, USA). The data of absorbance and curcumin concentration were plotted to generate a standard curve, and the regression equation, along with the correlation coefficient (R^2^), was calculated [39].

The release profiles of Mg^2+^ and curcumin from hydrogel, respectively, were evaluated [40,41]. Each hydrogel (diameter: 10 mm, height: 2 mm) was immersed in 3 mL of PBS and incubated at 37 °C. At designated time points (1, 3, 6, 10, 24, 36, and 48 h), the entire supernatant (3 mL) was collected, the supernatant was removed, and 3 mL fresh PBS was added instead. The concentration of Mg^2+^ in the samples from CS-MA-EMA-DMA-MgO and CS-MA-EMA-DMA-MgO-Cur disks was measured by ICP-OES (AVIO 500, PerkinElmer, Shelton, CT, USA), and absorbance was measured at 425 nm using a UV-Vis spectrophotometer (Cary 5000, Agilent Technologies, Santa Clara, CA, USA) to calculate the released curcumin concentration of CS-MA-DMA-EMA-MgO-Cur disks. Cumulative releases were calculated accordingly.

#### 4.2.8. Rheological Properties of the Hydrogel

A HAAKE RS6000 rheometer (Thermo Fisher Scientific, Karlsruhe, Germany) with a 25 mm parallel plate was used for rheological characterization at 25 °C. The hydrogels underwent two tests: frequency sweep and strain sweep. Frequency sweep was implemented over 1–50 Hz with 1% strain, and strain sweep was set within 0.1–1000% strain at 1 Hz. All experiments were conducted at 25 °C to evaluate hydrogel stability.

#### 4.2.9. Hemolysis Ratio (HR)

Hemolysis ratio (HR) of the sample surfaces was evaluated using rabbit whole blood treated with 3.2% sodium citrate for anticoagulation. Samples were first soaked in 1 mL 0.9% sodium chloride solution at 37 °C for 2 h. Subsequently, 200 μL whole blood was added, and the system was incubated for another 1 h at 37 °C. After centrifugation at 2000 rpm for 20 min, the absorbance of hemoglobin released from hemolyzed blood was measured at 545 nm [42]. HR values were computed via the corresponding formula, with normal saline as the negative control and pure water as the positive control:

(2) $$HR\left(\%\right)=\frac{AS-AN}{AP-AN}$$

where AN and AP denote the absorbance values of the negative and positive controls at 545 nm, and AS represents the absorbance of the sample supernatant measured at 545 nm. Each test was repeated five times (n = 5).

#### 4.2.10. In Vitro Antibacterial Activity

*E. coli* or *S. aureus* was cultivated in Luria–Bertani (LB) medium prepared with 10 mg/mL NaCl, 10 mg/mL tryptone (Oxoid, Basingstoke, Hampshire, UK), and 5 mg/mL yeast extract (Oxoid, Basingstoke, Hampshire, UK) at 37 °C. To prepare solid media, 15–20 mg/mL agar (Coolaber, Beijing, China) was incorporated, and the mixture was dispensed into plates for later use. A 10 μL portion of an overnight culture was transferred into 5 mL of fresh LB broth and incubated at 37 °C with agitation at 200 rpm until the optical density at 600 nm (OD600) reached approximately 0.5. The cells were collected by centrifugation at 6000 rpm, washed three times with 0.01 M phosphate-buffered saline (PBS), and resuspended in LB medium supplemented with the different test substances. Following a 12 h incubation, the suspensions were serially diluted, and 100 μL of a dilution yielding 10^3^ CFU/mL was spread onto LB agar plates. The plates were then incubated at 37 °C for 12 h, and bacterial viability was evaluated by the standard plate count method [43].

#### 4.2.11. Cell Viability

Cytotoxicity of the sample extracts was quantitatively evaluated using the 3-(4,5-dimethylthiazol-2-yl)-2,5-diphenyltetrazolium bromide (MTT) assay [44,45]. The extracts were prepared so as to mimic clinical exposure conditions without directly dissolving the test materials in culture medium. Subsequently, 100 µL of a cell suspension was seeded into 96-well plates at a density of 5 × 10^3^ cells per well and incubated for 4 h. The culture medium was then replaced with the extracts at various concentrations. After 1 and 3 days of incubation, 10 µL of an MTT solution (5 mg mL^−1^ in PBS; Sangon Biotech, Shanghai, China) was added to each well. Following a further 4 h incubation at 37 °C, the solution was carefully removed, and dimethyl sulfoxide (DMSO) was added to dissolve the formazan crystals. The absorbance of the resulting formazan product was measured at 570 nm using a microplate spectrophotometer (BioTek Epoch 2T; BioTek Instruments, Inc., Winooski, VT, USA) [46,47].

#### 4.2.12. Cell Proliferation

Cell proliferation was evaluated using the Cell Counting Kit-8 (CCK-8) assay (CA1210, Solarbio Science & Technology Co., Ltd., Beijing, China) following the manufacturer’s instructions. MC3T3 cells were seeded into 96-well plates and maintained in α-MEM for 1, 3, and 7 days. At each time point, 10 µL of CCK-8 reagent was added to each well, and the plates were incubated at 37 °C for 4 h. The absorbance was subsequently recorded at 450 nm with a microplate reader (Sangon Biotech Co., Ltd., Shanghai, China) [48,49,50].

#### 4.2.13. Flow Cytometry Assay

Flow cytometry was performed using antibodies against CD86 and CD206 to determine the proportion of M1 and M2 macrophages. Briefly, RAW264.7 macrophages were cultured with LPS (100 ng/mL) and hydrogel for 3 days. The cells were then stained with FITC-conjugated CD86 and APC-conjugated CD206 for 30 min at 4 °C in the dark. Finally, the cells were washed, resuspended in 500 µL of PBS, and analyzed by flow cytometry [51,52,53].

## Figures and Tables

**Table 1:** Nanoparticle size and potential of CS NPs and Cur-CS NPs.

Sample	Particle Size (nm)	Zeta-Potential (mV)
CS NPs	389.7 ± 44.6	5.2 ± 3.6
Cur-CS NPs	436.9 ± 115.7	9.7 ± 4.7

## Data Availability

Data are contained within the article and Supplementary Materials.

## Supplementary Materials

The following supporting information can be downloaded at: https://www.mdpi.com/article/10.3390/gels12070580/s1, Figure S1: MS spectrum of EMA; Figure S2: MS spectra of DMA; Figure S3: Synthesis of CS-MA; Figure S4: Adhesion characterization of hydrogel; Table S1: Pore size values of the hydrogels; Table S2: Composition of hydrogel formulations.

## Abbreviations

The following abbreviations are used in this manuscript:

EMA	Ectoine methacrylate
DMA	Dopamine methacrylamide
CS-MA	Methacrylated chitosan
Cur-CS NPs	Encapsulation of curcumin into chitosan nanoparticles
