# Supplementary material for: Chitosan-Based Adhesive Composite Hydrogel to Provide Antibacterial and Anti-Inflammatory Properties for Biomedical Application
Source: Gels. 2026 Jul 1;12(7):580. doi: 10.3390/gels12070580 (PMC13409680; doi:10.3390/gels12070580)
Supplement: Supplementary file 1 [file gels-12-00580-s001.zip › gels-4369905-supplementary.pdf]

SUPPLEMENTARY INFORMATION

Chitosan-Based Adhesive Composite Hydrogel to Provide Antibacterial and Anti-Inflammatory Properties for Biomedical Application

Sinuo Yan [1], Jingna Xu [2], Yue Shen [2], Chengde Liu [2,*], Xigao Jian [2] and Zhonghai Li [1,*]

[1] Dalian Medical University, Dalian 116000, China; yansinuodalian@outlook.com; lizhonghai@dmu.edu.cn

[2] Department of Polymer Science & Materials, Dalian University of Technology, Dalian, 116024, People's Republic of China. xujingna@mail.dlut.edu.cn; yueshen@dlut.edu.cn; liucd@dlut.edu.cn; jian4616@dlut.edu.cn

[*] Correspondence: lizhonghai@dmu.edu.cn; liucd@dlut.edu.cn

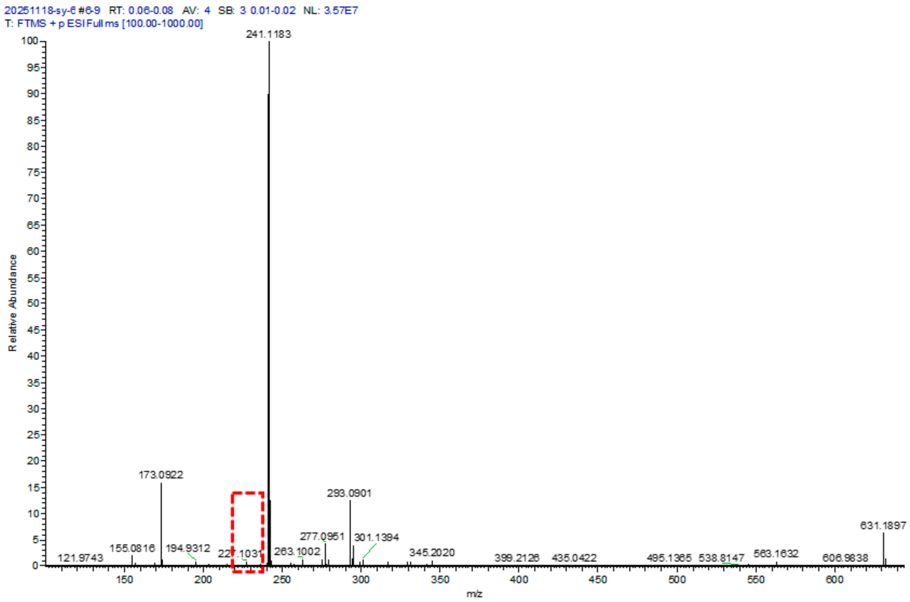

Figure S1. MS spectrum of EMA.

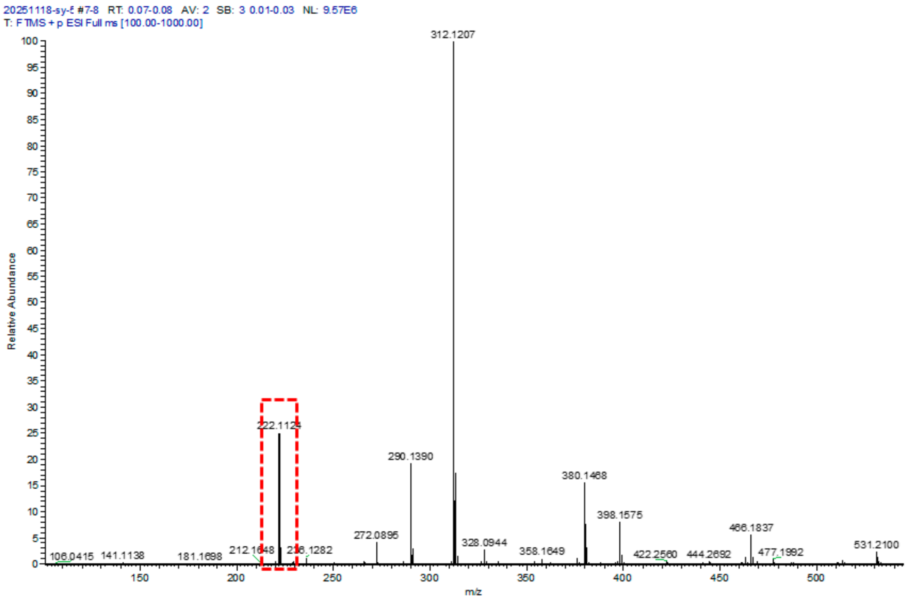

Figure S2. MS spectra of DMA.

Table S1. Pore size values of the hydrogels.

Sample	CS-MA	CS-MA-EMA	CS-MA-EMA-DMA	CS-MA-EMA-DMA-MgO	CS-MA-EMA-DMA-MgO-Cur
Pore size (μm)	47.423	38.165	36.639	21.678	13.247

Table S2. Composition of hydrogel formulations.

Sample	CS-MA	EMA	DMA	MgO	Cur-CS
Content	10 wt%	8wt%	0.2 wt%	0.2 wt%	0.25 mg/mL

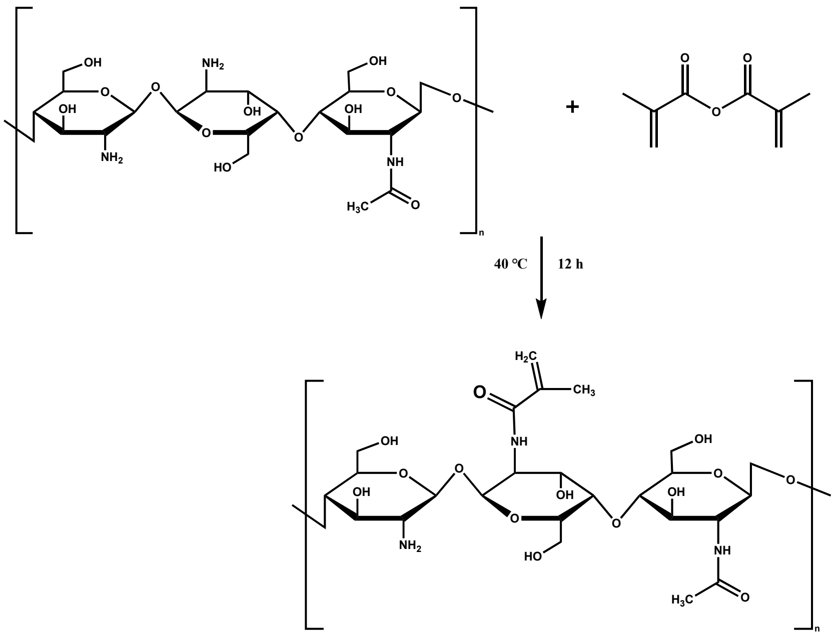

Figure S3. Synthesis of CS-MA.

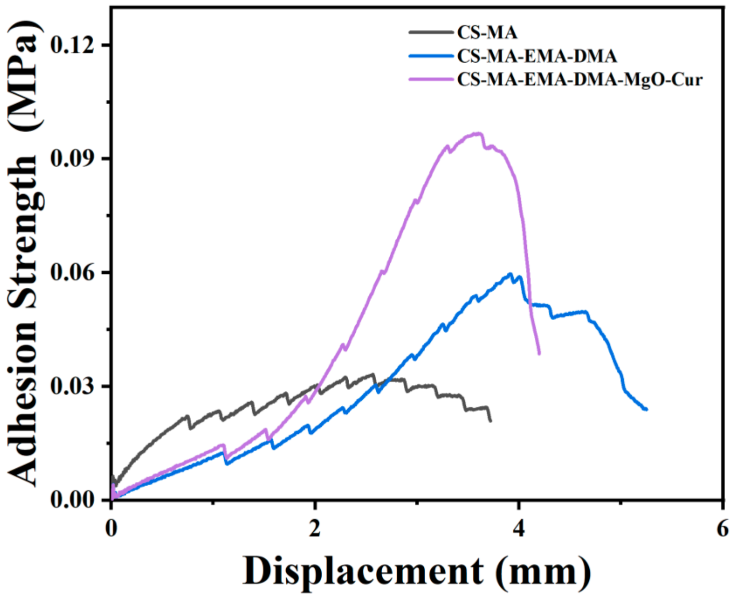

Figure S4. Adhesion characterization of hydrogel.
